# Coronary remodeling and stiffness in older hypertensive patients: an MR imaging study

**DOI:** 10.1186/1532-429X-14-S1-P171

**Published:** 2012-02-01

**Authors:** Kai Lin, Donald M Lloyd-Jones, Ying Liu, Xiaoming Bi, Debiao Li, James Carr

**Affiliations:** 1 Radiology, Northwestern University, Chicago, IL, USA; 2 Siemens Healthcare, Chicago, IL, USA

## Summary

There are interrelations among coronary stiffness, wall thickening and remodeling pattern in older hypertensive patients. MR techniques are able to provide both the morphology and the biomechanic information of the coronary wall in a single scan.

## Background

Coronary artery remodeling is an indicator of coronary artery disease. Morphological and biomechanical changes accompany with coronary wall remodeling. Such changes can be noninvasively detected using MR imaging and may be used to predict cardiovascular events. The aim of this study is to assess interrelations among various imaging markers of the coronary remodeling in older hypertensive patients.

## Methods

This study was approved by the institutional review board. Two-dimensional black-blood coronary wall MR imaging and three-dimensional whole-heart coronary MR angiography (imaging data acquired in both systole and in diastole) were performed on 65 asymptomatic hypertensive patients (73.4 years ± 5.5). Coronary vessel area, wall area, lumen area, wall thickness were measured. The percent of the coronary wall occupying the vessel area (PWOV) (defined as [Vessel wall area / Vessel area x 100%]) and coronary distensibility index (CDI) (defined as [(Lumen systolic - Lumen diastolic) / (Lumen diastolic x Pulse pressure)] x 1000) were calculated . Transverse coronary segments were assigned to two groups using mean PWOV as an ad hoc cutoff point. Coronary indices were compared between the two groups.

## Results

Totally 259 coronary segments were eligible for analysis (mean PWOV 74.5%). The CDI (5.30 ± 2.60 mmHg-1) was significantly correlated with mean wall thickness(1.43 ± 0.26 mm, r = 0.541), max wall thickness (1.92 ± 0.33 mm, r = 0.503). The PWOV was significantly correlated with mean wall thickness (r = 0.647), max wall thickness (r = 0.603) and lumen area (6.57 ± 3.44 mm2, r = 0.796). One hundred and forty segments (group 1) had PWOVs higher than 74.5%, while 119 segments (group 2) had PWOV lower than 74.5%. Segments in group2 had a significantly lower mean (1.29 ± 0.22 mm vs. 1.54 ± 0.23 mm, P < 0.001) and max wall thickness (1.78 ± 0.30 mm vs. 2.05 ± 0.31 mm, P < 0.001), a larger vessel area (27.43 ± 8.39mm2 vs. 23.05 ± 6.35 mm2, P < 0.001), a larger lumen area (9.04 ± 3.34 mm2 vs. 4.46 ± 1.68 mm2, P < 0.001) and a higher CDI (5.89 ± 2.65 mmHg-1 vs. 4.79 ± 2.46 mmHg-1, P = 0.001) compared with those of segments in group 1. For the 10 randomly chosen cases, good intra-observer (r = 0.866 for CDI and r = 0.911 for wall thickness) and inter-observer agreement (r = 0.812 for CDI and r = 0.898 for wall thickness) was found on 43 coronary segments. The scan-rescan test showed low variation between coronary measurements in 38 coronary segments from the 10 randomly chosen cases (r = 0.751 for CDI and r = 0.816 for wall thickness).

**Figure 1:**
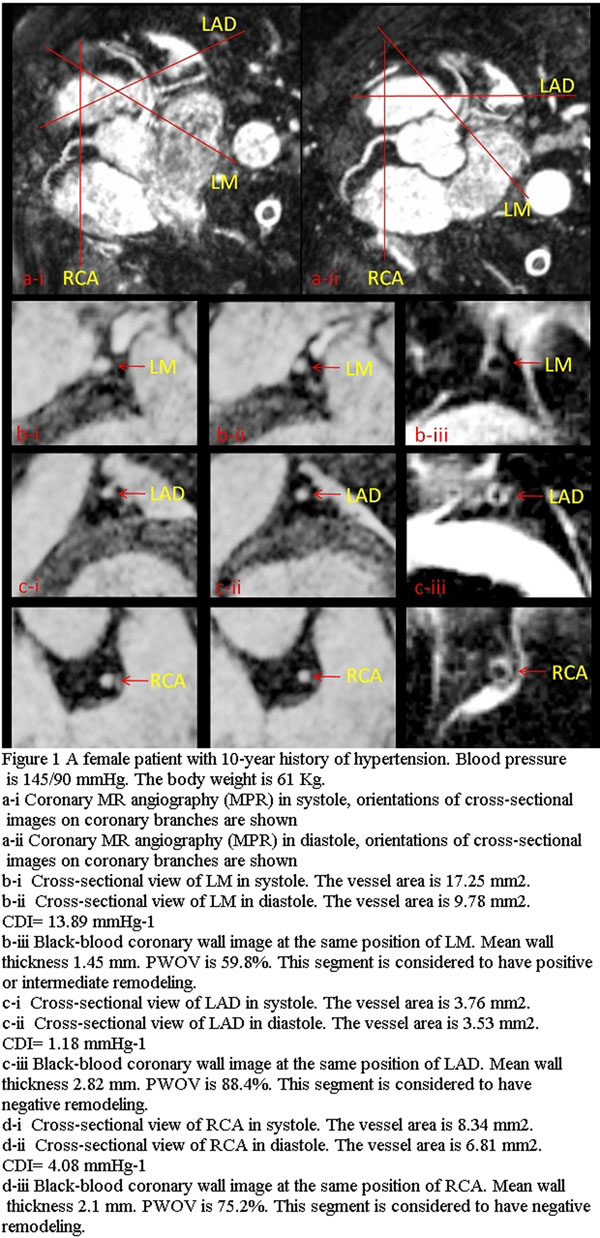
A female patient with 10-year history of hypertension.

A set of images for coronary segments with different PWOV from a single patient is shown in Figure 1.

## Conclusions

Aterial stiffness is correlated with wall thickness of the remodeled coronary artery in older hypertensive patients. Reflecting relaions between wall thickening and lumen area variation, PWOV has the potential to become a quantitative index of coronary remodeling patterns (such as positive or negative remodeling) in imaging studies.

## Funding

National Institute of Health (R01HL089695)

American Heart Association (10CRP3050051)

**Table 1:** Basic information of participants in present study

Age (years ± SD) 73.4 ± 5.5
Male (age ± SD) 38 (74.0 ± 5.7)
Female (age ± SD) 27 (72.6 ± 5.2)
Heart rate (beats/minute ± SD) 61 ± 7.1
Diabetes (%) 17 (26.2)
SBP (mmHg) 140.0 ± 17.3
DBP (mmHg) 85.2 ± 13.8
PP (mmHg) 54.7 ± 8.6
BP under control (%) 59 (90.7) Page 22 of 33 Prudential

